# 5-Hy­droxy-2-{(*E*)-[(3-nitro­phen­yl)iminio]meth­yl}phenolate

**DOI:** 10.1107/S1600536812033740

**Published:** 2012-08-01

**Authors:** Muhammad Ashraf Shaheen, M. Nawaz Tahir, Rana Muhammad Irfan, Shahid Iqbal, Saeed Ahmad

**Affiliations:** a University of Sargodha, Department of Chemistry, Sargodha, Pakistan; b University of Sargodha, Department of Physics, Sargodha, Pakistan; c University of Engineering and Technology, Department of Chemistry, Lahore 54890, Pakistan

## Abstract

The title compound, C_13_H_10_N_2_O_4_, crystallized as the zwitterionic tautomer. As a result, the phenolate C—O^−^ bond [1.296 (2) Å] is shorter than a normal C*sp* ^2^—O(H) bond, and the azomethine C=N bond [1.314 (2) Å] is longer than a normal C=N double bond. The mol­ecule is nearly planar, the mean plane of the nitro-substituted benzene ring forming dihedral angles of 9.83 (7) and 8.45 (9)° with the other benzene ring and with the nitro group, respectively. The mol­ecular conformation is stabilized by an intra­molecular N—H⋯O hydrogen bond. In the crystal, strong O—H⋯O hydrogen bonds link the mol­ecules into double-stranded chains along the *b*-axis direction. Within the chains there are π–π interactions involving the benzene rings of adjacent molecules [centroid–centroid distance = 3.669 (1) Å]. The chains are linked *via* C—H⋯O hydrogen bonds, forming *R*_2_^1^(6), *R*_2_^1^(7) and *R*_2_^2^(10) ring motifs.

## Related literature

For related structures, see: Yeap *et al.* (1992 ▶); Hijji *et al.* (2009 ▶). For graph-set analysis of hydrogen bonds, see: Bernstein *et al.* (1995 ▶).
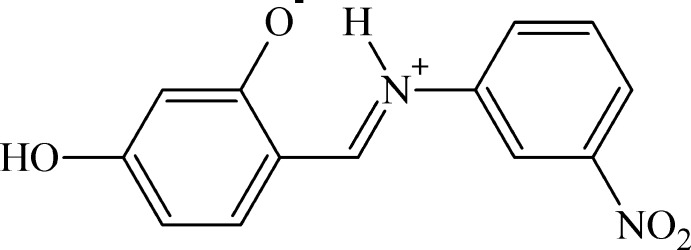


## Experimental

### 

#### Crystal data

C_13_H_10_N_2_O_4_
*M* *_r_* = 258.23
Monoclinic, 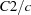

*a* = 12.8518 (9) Å
*b* = 7.8501 (5) Å
*c* = 24.1316 (18) Å
β = 101.593 (3)°
*V* = 2384.9 (3) Å^3^
*Z* = 8
Mo *K*α radiation
μ = 0.11 mm^−1^
*T* = 296 K
0.30 × 0.25 × 0.22 mm

#### Data collection

Bruker Kappa APEXII CCD area-detector diffractometer
Absorption correction: multi-scan (*SADABS*; Bruker, 2009 ▶) *T*_min_ = 0.975, *T*_max_ = 0.985
5601 measured reflections
2126 independent reflections
1569 reflections with *I* > 2σ(*I*)
*R*_int_ = 0.025

#### Refinement

*R*[*F* ^2^ > 2σ(*F* ^2^)] = 0.038
*wR*(*F* ^2^) = 0.105
*S* = 1.02
2126 reflections
173 parameters
H-atom parameters constrained
Δρ_max_ = 0.13 e Å^−3^
Δρ_min_ = −0.15 e Å^−3^

### 

Data collection: *APEX2* (Bruker, 2009 ▶); cell refinement: *SAINT* (Bruker, 2009 ▶); data reduction: *SAINT*; program(s) used to solve structure: *SHELXS97* (Sheldrick, 2008 ▶); program(s) used to refine structure: *SHELXL97* (Sheldrick, 2008 ▶); molecular graphics: *ORTEP-3 for Windows* (Farrugia, 1997 ▶) and *PLATON* (Spek, 2009 ▶); software used to prepare material for publication: *WinGX* (Farrugia, 1999 ▶) and *PLATON*.

## Supplementary Material

Crystal structure: contains datablock(s) global, I. DOI: 10.1107/S1600536812033740/yk2068sup1.cif

Structure factors: contains datablock(s) I. DOI: 10.1107/S1600536812033740/yk2068Isup2.hkl

Supplementary material file. DOI: 10.1107/S1600536812033740/yk2068Isup3.cml

Additional supplementary materials:  crystallographic information; 3D view; checkCIF report

## Figures and Tables

**Table 1:** Hydrogen-bond geometry (Å, °)

*D*—H⋯*A*	*D*—H	H⋯*A*	*D*⋯*A*	*D*—H⋯*A*
N2—H2*A*⋯O3	0.86	1.87	2.5716 (19)	138
O4—H4*A*⋯O3^i^	0.82	1.79	2.6100 (17)	179
C2—H2⋯O2^ii^	0.93	2.54	3.446 (2)	164
C4—H4⋯O4^iii^	0.93	2.54	3.268 (2)	135
C7—H7⋯O2^ii^	0.93	2.49	3.355 (2)	154
C10—H10⋯O3^i^	0.93	2.56	3.226 (2)	129

Symmetry codes: (i) 
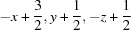
; (ii) 
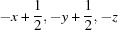
; (iii) 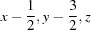.

**Fig. 1.**
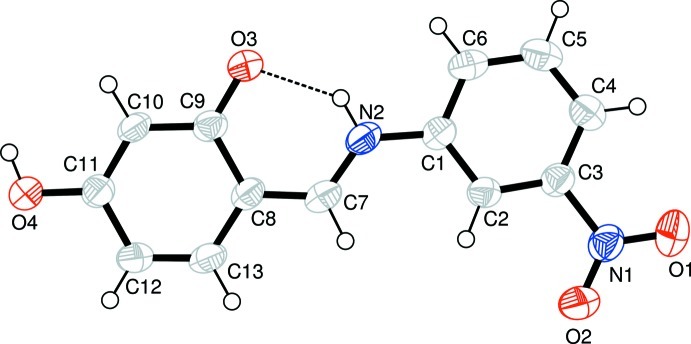
Molecular structure of the title compound with the atom-numbering scheme. The thermal ellipsoids are drawn at the 50% probability level.

**Fig. 2.**
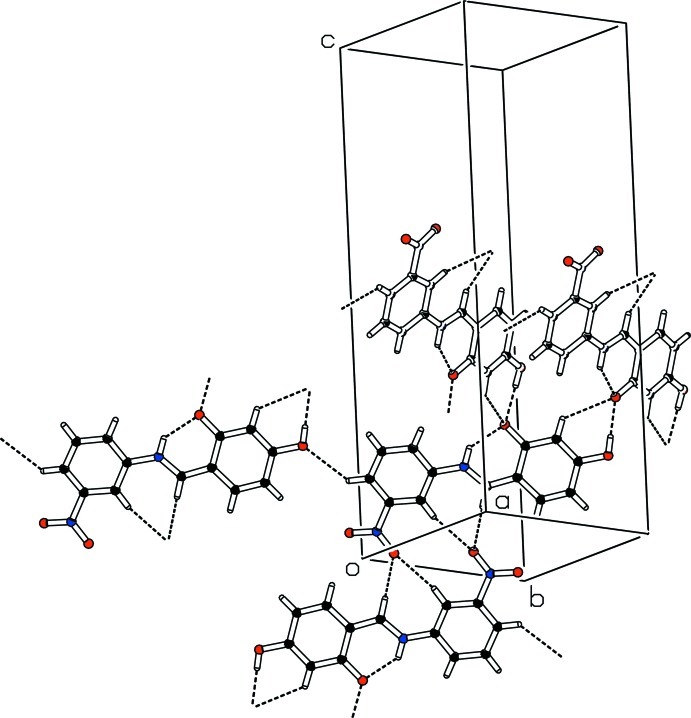
The packing diagram showing the chains along the [010] direction and various ring motifs.

## supplementary crystallographic information

### Comment

The title compound (Fig. 1) has been synthesized as a precursor for complex formation and other studies.

In contrast to the closely related structure of 2-[(3-nitrophenylimino)methyl]phenol (Yeap *et al.*, 1992), the title compound is a zwitterion, in which the hydroxy H^+^ ion is transferred to the imino N atom (Fig. 1). Analogous zwitterionic structure is observed for 2-{[(2-hydroxy-5-nitrophenyl)iminio]methyl}phenolate (Hijji *et al.*, 2009).

The molecule consists of two roughly planar groups, the 3-nitroaniline fragment (C1—C6/N1/N2/O1/O2) and the rest of 2,4-dihydroxybenzaldehyde (C7—C13/O3/O4), the mean deviations from the planes are 0.070Å and 0.023Å, respectively. The dihedral angle between the planes of these groups is 9.37 (6)°.

Strong intramolecular N—H···O hydrogen bond (Table 1, Fig. 2) produce *S*(6) ring motif (Bernstein *et al.*, 1995). Due to the intermolecular O—H···O hydrogen bonds, the *C*(6) chains along the *b*-axis direction are formed (Table 1, Fig. 2). The C—H···O interactions join these chains, generating the *R*_2_^1^(7) and *R*_2_^2^(10) rings. motifs. Due to the C—H···O and O—H···O hydrogen bonds, the *R*_2_^1^(6) ring motif is also formed (Table 1, Fig. 2).

### Experimental

3-Nitroaniline (0.138 g, 1.0 mmol) was dissolved in distilled methanol. Solution of 2,4-dihydroxybenzaldehyde (0.138 g, 1.0 mmol) in methanol was added dropwise. The mixture was refluxed for 2 h and orange prisms of the title compound were obtained after 48 h.

### Refinement

At initial stages, all H atoms were refined freely, indicating the zwitterion structure. Later, all H atoms were positioned geometrically at C—H = 0.93, N—H = 0.86 and O—H = 0.82 Å, respectively, and refined as riding with *U*_iso_(H) = *xU*_eq_(C, N, O), where *x* = 1.5 for hydroxy and *x* = 1.2 for other H atoms.

### Crystal data

**Table:**

C_13_H_10_N_2_O_4_	*F*(000) = 1072
*M**_r_* = 258.23	*D*_x_ = 1.438 Mg m^−^^3^
Monoclinic, *C*2/*c*	Mo *K*α radiation, λ = 0.71073 Å
Hall symbol: -C 2yc	Cell parameters from 1569 reflections
*a* = 12.8518 (9) Å	θ = 3.1–25.3°
*b* = 7.8501 (5) Å	µ = 0.11 mm^−^^1^
*c* = 24.1316 (18) Å	*T* = 296 K
β = 101.593 (3)°	Prism, orange
*V* = 2384.9 (3) Å^3^	0.30 × 0.25 × 0.22 mm
*Z* = 8	

### Data collection

**Table:**

Bruker Kappa APEXII CCD area-detector diffractometer	2126 independent reflections
Radiation source: fine-focus sealed tube	1569 reflections with *I* > 2σ(*I*)
Graphite monochromator	*R*_int_ = 0.025
Detector resolution: 8.10 pixels mm^-1^	θ_max_ = 25.3°, θ_min_ = 3.1°
ω scans	*h* = −15→15
Absorption correction: multi-scan (*SADABS*; Bruker, 2009)	*k* = −9→8
*T*_min_ = 0.975, *T*_max_ = 0.985	*l* = −28→27
5601 measured reflections	

### Refinement

**Table:**

Refinement on *F*^2^	Primary atom site location: structure-invariant direct methods
Least-squares matrix: full	Secondary atom site location: difference Fourier map
*R*[*F*^2^ > 2σ(*F*^2^)] = 0.038	Hydrogen site location: inferred from neighbouring sites
*wR*(*F*^2^) = 0.105	H-atom parameters constrained
*S* = 1.02	*w* = 1/[σ^2^(*F*_o_^2^) + (0.0478*P*)^2^ + 0.8634*P*] where *P* = (*F*_o_^2^ + 2*F*_c_^2^)/3
2126 reflections	(Δ/σ)_max_ < 0.001
173 parameters	Δρ_max_ = 0.13 e Å^−^^3^
0 restraints	Δρ_min_ = −0.15 e Å^−^^3^

### Special details

**Table:**

Geometry. Bond distances, angles *etc*. have been calculated using the rounded fractional coordinates. All su's are estimated from the variances of the (full) variance-covariance matrix. The cell e.s.d.'s are taken into account in the estimation of distances, angles and torsion angles
Refinement. Refinement of *F*^2^ against ALL reflections. The weighted *R*-factor *wR* and goodness of fit *S* are based on *F*^2^, conventional *R*-factors *R* are based on *F*, with *F* set to zero for negative *F*^2^. The threshold expression of *F*^2^ > σ(*F*^2^) is used only for calculating *R*-factors(gt) *etc*. and is not relevant to the choice of reflections for refinement. *R*-factors based on *F*^2^ are statistically about twice as large as those based on *F*, and *R*- factors based on ALL data will be even larger.

### Fractional atomic coordinates and isotropic or equivalent isotropic displacement parameters (Å^2^)

**Table:**

	*x*	*y*	*z*	*U*_iso_*/*U*_eq_	
O1	0.15456 (11)	−0.19142 (18)	0.02818 (6)	0.0662 (5)	
O2	0.20746 (12)	0.03661 (18)	−0.00681 (6)	0.0724 (6)	
O3	0.62612 (10)	0.43187 (15)	0.22353 (5)	0.0534 (4)	
O4	0.76284 (11)	0.95669 (16)	0.17361 (5)	0.0575 (5)	
N1	0.21457 (12)	−0.0694 (2)	0.03061 (7)	0.0496 (6)	
N2	0.49661 (11)	0.26941 (19)	0.14644 (6)	0.0474 (5)	
C1	0.43511 (13)	0.1203 (2)	0.13474 (7)	0.0410 (6)	
C2	0.35653 (14)	0.1006 (2)	0.08681 (7)	0.0421 (6)	
C3	0.29944 (13)	−0.0494 (2)	0.08125 (7)	0.0414 (6)	
C4	0.31558 (15)	−0.1781 (2)	0.12040 (8)	0.0481 (6)	
C5	0.39528 (16)	−0.1569 (3)	0.16724 (8)	0.0530 (7)	
C6	0.45478 (14)	−0.0106 (3)	0.17409 (7)	0.0487 (6)	
C7	0.50024 (13)	0.4025 (2)	0.11385 (7)	0.0447 (6)	
C8	0.56478 (13)	0.5432 (2)	0.13102 (7)	0.0405 (6)	
C9	0.62972 (13)	0.5521 (2)	0.18714 (7)	0.0403 (6)	
C10	0.69554 (13)	0.6942 (2)	0.20049 (7)	0.0406 (6)	
C11	0.69865 (13)	0.8209 (2)	0.16195 (7)	0.0416 (6)	
C12	0.63360 (14)	0.8146 (2)	0.10705 (7)	0.0461 (6)	
C13	0.56882 (14)	0.6792 (2)	0.09292 (7)	0.0459 (6)	
H2	0.34268	0.18528	0.05936	0.0505*	
H2A	0.53731	0.27396	0.17938	0.0569*	
H4	0.27414	−0.27611	0.11547	0.0578*	
H4A	0.79725	0.94912	0.20604	0.0862*	
H5	0.40893	−0.24223	0.19446	0.0636*	
H6	0.50928	0.00111	0.20566	0.0584*	
H7	0.45787	0.40280	0.07774	0.0536*	
H10	0.73819	0.70276	0.23640	0.0487*	
H12	0.63551	0.90201	0.08126	0.0553*	
H13	0.52555	0.67518	0.05708	0.0551*	

### Atomic displacement parameters (Å^2^)

**Table:**

	*U*^11^	*U*^22^	*U*^33^	*U*^12^	*U*^13^	*U*^23^
O1	0.0630 (9)	0.0603 (9)	0.0709 (10)	−0.0201 (8)	0.0033 (7)	−0.0121 (7)
O2	0.0882 (12)	0.0606 (9)	0.0540 (9)	−0.0102 (8)	−0.0198 (8)	0.0103 (8)
O3	0.0592 (8)	0.0484 (7)	0.0437 (8)	−0.0070 (6)	−0.0110 (6)	0.0081 (6)
O4	0.0692 (9)	0.0485 (8)	0.0460 (8)	−0.0139 (7)	−0.0092 (6)	0.0048 (6)
N1	0.0527 (10)	0.0446 (9)	0.0478 (10)	−0.0009 (8)	0.0010 (7)	−0.0077 (8)
N2	0.0451 (9)	0.0506 (9)	0.0399 (9)	−0.0003 (8)	−0.0072 (6)	−0.0014 (7)
C1	0.0396 (10)	0.0429 (10)	0.0386 (10)	0.0018 (8)	0.0037 (7)	−0.0030 (8)
C2	0.0469 (10)	0.0370 (10)	0.0389 (10)	0.0026 (8)	0.0002 (8)	0.0031 (7)
C3	0.0418 (10)	0.0400 (10)	0.0401 (10)	0.0022 (8)	0.0031 (8)	−0.0036 (8)
C4	0.0514 (11)	0.0426 (10)	0.0510 (11)	0.0008 (9)	0.0117 (9)	0.0057 (9)
C5	0.0567 (12)	0.0538 (12)	0.0484 (12)	0.0081 (10)	0.0103 (9)	0.0167 (9)
C6	0.0469 (11)	0.0619 (12)	0.0347 (10)	0.0056 (10)	0.0023 (8)	0.0053 (9)
C7	0.0391 (10)	0.0532 (11)	0.0374 (10)	0.0041 (9)	−0.0025 (8)	−0.0024 (9)
C8	0.0368 (9)	0.0432 (10)	0.0378 (10)	0.0029 (8)	−0.0016 (7)	−0.0044 (8)
C9	0.0391 (10)	0.0399 (10)	0.0385 (10)	0.0062 (8)	−0.0002 (7)	0.0001 (8)
C10	0.0414 (10)	0.0429 (10)	0.0318 (9)	0.0022 (8)	−0.0059 (7)	−0.0033 (8)
C11	0.0429 (10)	0.0391 (10)	0.0403 (10)	0.0018 (8)	0.0024 (8)	−0.0024 (8)
C12	0.0514 (11)	0.0479 (11)	0.0356 (10)	0.0027 (9)	0.0007 (8)	0.0050 (8)
C13	0.0462 (11)	0.0528 (11)	0.0337 (10)	0.0048 (9)	−0.0040 (8)	0.0004 (8)

### Geometric parameters (Å, º)

**Table:**

O1—N1	1.224 (2)	C7—C8	1.393 (2)
O2—N1	1.218 (2)	C8—C13	1.417 (2)
O3—C9	1.296 (2)	C8—C9	1.443 (2)
O4—C11	1.343 (2)	C9—C10	1.398 (2)
O4—H4A	0.8200	C10—C11	1.368 (2)
N1—C3	1.474 (2)	C11—C12	1.418 (2)
N2—C7	1.314 (2)	C12—C13	1.351 (2)
N2—C1	1.409 (2)	C2—H2	0.9300
N2—H2A	0.8600	C4—H4	0.9300
C1—C6	1.388 (3)	C5—H5	0.9300
C1—C2	1.383 (2)	C6—H6	0.9300
C2—C3	1.380 (2)	C7—H7	0.9300
C3—C4	1.370 (2)	C10—H10	0.9300
C4—C5	1.375 (3)	C12—H12	0.9300
C5—C6	1.371 (3)	C13—H13	0.9300
			
C11—O4—H4A	109.00	C8—C9—C10	117.65 (14)
O1—N1—O2	123.14 (17)	C9—C10—C11	121.51 (15)
O1—N1—C3	118.52 (15)	O4—C11—C12	116.45 (14)
O2—N1—C3	118.35 (15)	O4—C11—C10	122.38 (15)
C1—N2—C7	128.80 (15)	C10—C11—C12	121.17 (15)
C7—N2—H2A	116.00	C11—C12—C13	118.74 (15)
C1—N2—H2A	116.00	C8—C13—C12	122.02 (16)
N2—C1—C2	123.13 (15)	C1—C2—H2	121.00
N2—C1—C6	117.42 (15)	C3—C2—H2	121.00
C2—C1—C6	119.45 (16)	C3—C4—H4	121.00
C1—C2—C3	117.49 (15)	C5—C4—H4	121.00
N1—C3—C2	117.52 (14)	C4—C5—H5	120.00
N1—C3—C4	118.52 (15)	C6—C5—H5	120.00
C2—C3—C4	123.95 (16)	C1—C6—H6	119.00
C3—C4—C5	117.53 (17)	C5—C6—H6	119.00
C4—C5—C6	120.39 (19)	N2—C7—H7	119.00
C1—C6—C5	121.15 (16)	C8—C7—H7	119.00
N2—C7—C8	122.88 (15)	C9—C10—H10	119.00
C7—C8—C13	120.11 (15)	C11—C10—H10	119.00
C7—C8—C9	121.00 (15)	C11—C12—H12	121.00
C9—C8—C13	118.88 (15)	C13—C12—H12	121.00
O3—C9—C8	120.53 (14)	C8—C13—H13	119.00
O3—C9—C10	121.82 (15)	C12—C13—H13	119.00
			
O1—N1—C3—C2	−171.10 (16)	C4—C5—C6—C1	1.1 (3)
O1—N1—C3—C4	7.6 (2)	N2—C7—C8—C9	−1.9 (3)
O2—N1—C3—C2	8.8 (2)	N2—C7—C8—C13	177.02 (16)
O2—N1—C3—C4	−172.56 (17)	C7—C8—C9—O3	−2.7 (3)
C7—N2—C1—C2	−8.0 (3)	C7—C8—C9—C10	177.49 (16)
C7—N2—C1—C6	172.80 (17)	C13—C8—C9—O3	178.36 (16)
C1—N2—C7—C8	179.59 (16)	C13—C8—C9—C10	−1.5 (2)
N2—C1—C2—C3	−177.80 (16)	C7—C8—C13—C12	−177.19 (17)
C6—C1—C2—C3	1.4 (3)	C9—C8—C13—C12	1.8 (3)
N2—C1—C6—C5	177.00 (17)	O3—C9—C10—C11	−179.76 (16)
C2—C1—C6—C5	−2.2 (3)	C8—C9—C10—C11	0.1 (2)
C1—C2—C3—N1	179.08 (15)	C9—C10—C11—O4	−178.67 (16)
C1—C2—C3—C4	0.5 (3)	C9—C10—C11—C12	1.1 (3)
N1—C3—C4—C5	179.88 (17)	O4—C11—C12—C13	178.96 (16)
C2—C3—C4—C5	−1.6 (3)	C10—C11—C12—C13	−0.9 (3)
C3—C4—C5—C6	0.7 (3)	C11—C12—C13—C8	−0.6 (3)

### Hydrogen-bond geometry (Å, º)

**Table:**

*D*—H···*A*	*D*—H	H···*A*	*D*···*A*	*D*—H···*A*
N2—H2*A*···O3	0.86	1.87	2.5716 (19)	138
O4—H4*A*···O3^i^	0.82	1.79	2.6100 (17)	179
C2—H2···O2^ii^	0.93	2.54	3.446 (2)	164
C4—H4···O4^iii^	0.93	2.54	3.268 (2)	135
C7—H7···O2^ii^	0.93	2.49	3.355 (2)	154
C10—H10···O3^i^	0.93	2.56	3.226 (2)	129

Symmetry codes: (i) −*x*+3/2, *y*+1/2, −*z*+1/2; (ii) −*x*+1/2, −*y*+1/2, −*z*; (iii) *x*−1/2, *y*−3/2, *z*.
